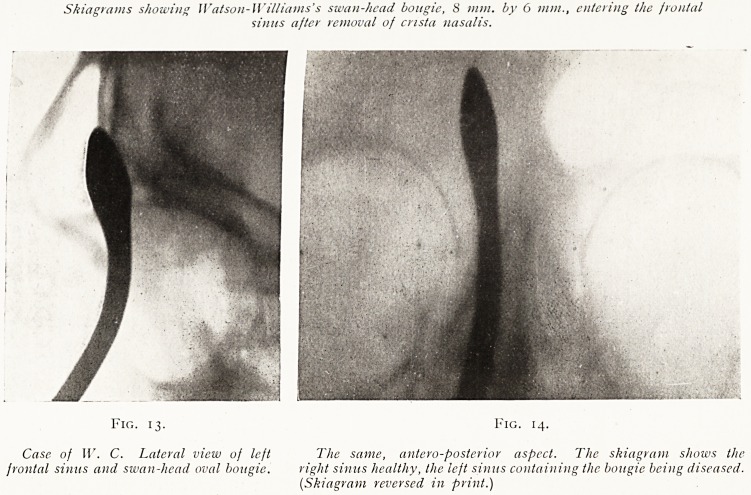# On the Pernasal Operation for Frontal Sinus Suppuration

**Published:** 1915-03

**Authors:** P. Watson-Williams

**Affiliations:** Lecturer on Otology, Rhinology, and Laryngology, University of Bristol, and in charge of the Department for Diseases of the Ear, Nose and Throat, Bristol Royal Infirmary


### ON THE PERNASAL OPERATION FOR FRONTAL SINUS SUPPURATION.

BY

P. Watson-Williams, M.D.Lond.,

*Lecturer on Otology, Rhinology, and Laryngology, University of Bristol, and in charge of the Department for Diseases of the Ear, Nose and Throat, Bristol Royal Infirmary.*

Many patients on whom the writer's intranasal or pernasal operation has been performed for the relief of frontal sinus suppuration have been demonstrated at meetings of the

FIG. 2.

*Bone specimen showing the fronto-nasal passage with probe passing through; the dotted line shows the route of anterior entry through agger and other ante-conchal cells. Note the "anterior entry" is well in front of the cribriform plate.*

Bristol Medico-Chirurgical Society, and I propose to describe shortly the operative technique of what I have termed the anterior route, without touching on the various methods of intranasal operation employed by other workers, which have been incorporated in my Introduction to a recent discussion on this subject at the Royal Society of Medicine.[1]

The non-external operations for frontal sinusitis fall under two classes, (*a*) those restricted to the removal of ethmoid cells and other structures within the nasal fossa below the frontal sinus, *i.e.* the strictly intranasal operations; and (*b*) those in which the operative field comprises parts entering into the formation of the sinus itself, *e.g.* the nasal crest and any other structures above the lower end of the ostium frontale, in which case the operation is no longer intranasal, but pernasal. The terms *intranasal* and *pernasal*[2] are, I

FIG. 3.
*The author's spheno-ethmoidal angular forceps, made in two sizes. All the instruments are marked in inches from the tip, so that the precise distance from the naris that any instrument has been passed can be at once determined.*

think, better terms for the difference in extent of operation than the words "incomplete" and "complete," which I have formerly used.

Practically all the internal operations for frontal sinus disease in general use are various degrees of intranasal

[1] *Proc. Roy. Soc. Med.*, 1914, ii. 121–43.
[2] The term *pernasal* was suggested to me by Mr. William Hill.

operation. To Fletcher Ingals, of Chicago, the credit is due of having devised the first practical method of operating on the frontal sinus ostium itself, *i.e.* of extending the operative field to the frontal sinus. Ingals reported in 1905 successful results from the operation, but it seemed to involve so much

FIG. 4.

*The author's guarded rotating burrs, which are fitted with a standard No. 2 Asch's "slip-on joint." The burr having been passed up the nose to the seat of obstruction, the nasal crest, the straight indicator, which is movable, is made to lie against the face outside and its tip indicates the position of the burr in situ. A finger disconnecter, not shown, allows the cable to be disconnected at any moment.*

risk to the patient that his method has never been generally accepted. Others have tried to improve on the methods of Ingals, and for some years I have sought what I venture to think are methods which at least have the advantage of being safe, provided reasonable care and skill be conjoined with an intimate practical acquaintance with the anatomy of the region and its variations from what is regarded as normal.

The alternative to intranasal or pernasal operative measures for frontal sinus suppuration is the external operation, which unfortunately affords no certainty of a completely successful result, always exposes the patient to the risk of septic osteo-myelitis, which is usually fatal, and is liable to leave a depressed scar, which may amount to a cosmetic deformity. Though in the course of a fairly large series of external operations I have been fortunate in having no case of osteo-myelitis, and the majority have been cured with little and sometimes no cosmetic defect, such happy results are far from invariable, and in two cases a fatal termination resulted, once from a congenital dehiscence in the cribriform plate, and in another patient from a pre-existing frontal lobe abscess.

An external radical frontal sinus operation should be avoided unless the symptoms are so severe that relief is urgently called for, and unless intranasal operations have failed or are impossible to perform. The intranasal or per-nasal method is sometimes rendered inapplicable owing to anatomical irregularities, which prevent one making a safe entrance to the sinus, and if no *entrance* can be effected, it is impossible to *enlarge* the fronto-nasal passage. I have met with one such case, and subsequently an external operation showed clearly that no operation through the nose could have succeeded. But, on the other hand, even a very large fronto-nasal passage, such as affords ample and effective drainage between the sinus and the nasal passages, does not ensure a cure or even satisfactory relief, for the sinus may be full of polypoid granulations, or may be in communication by a narrow opening with some large orbito-ethmoidal cell, which is altogether out of the reach of any pernasal operation. Yet such conditions are exceptional, and by the method described below the large majority of cases of frontal sinus suppuration, over 90 per cent., may be either cured or so greatly relieved that there is no need to consider a radical external operation.

An acute frontal sinusitis will often recover spontaneously, but chronic frontal sinusitis rarely, and for the reason that if the existing anatomical conditions or associated ethmoidal cell infection did not make a spontaneous recovery out of the question, the frontal sinus would have recovered in the early or acute stage, and thus would not have become chronic. It is therefore hardly

*Diagram of the ethmoidal labyrinth and fronto-ethmoidal cells, showing the curved entry, A to *, of the old method of catheterization of the frontal sinus, as compared with the direct anterior method of entry advocated, D to *—i.e., the creation of a new artificial fronto-nasal passage. A, A, agger cells; PU, plate of the uncinate process; PB, plate of the bulla, B; PMT, plate of the middle concha; PC''' and PC'''', plates of the concha superior and concha suprema.*

surprising that the intranasal treatment of chronic frontal sinusitis should have presented many difficulties, because almost invariably there are anatomical obstructions to entering the sinus, which have been the determining factor in preventing the draining of the sinus discharge. True, in a few cases, after an anterior middle turbinectomy the simple removal of a few of the lower ethmoidal cells around the lower end of the fronto-nasal passage suffices to permit of lavage of the sinus. But the infection of the higher and anterior ethmoidal cells often cause so much thickening of the mucosa and encroachment on the narrow passage, that no effective spontaneous drainage can result, and constantly-recurring acute attacks, or at any rate a persistence of the sinus disease, is then a frequent experience. To overcome the inherent defects of the accepted minor operative measures, a number of pernasal operations have been introduced, but for the most part have seemed to expose the patient to greater risks than the external radical operations which have therefore been very generally performed.

With a view to procuring spontaneous free drainage of the frontal sinus without danger, I devised the methods of procedure described below, and have operated on more than a hundred frontal sinuses of patients. The operation is usually a relatively easy one, and may be done under local anæsthesia, although for many patients a general anæsthetic is to be preferred.

It is always most desirable to have good skiagrams, one antero-posterior to show the extent of the sinuses upwards and laterally, and another lateral skiagram to show the depth of the sinuses. The importance of the skiagram as an aid to operation, apart from its value in the diagnosis of frontal sinus disease, lies in the fact that a quite small undeveloped frontal sinus may exist, and the instruments might reach the summit before they had apparently been properly introduced, and anyhow a small sinus presents more difficulties to pernasal operation than a large one. Of greater importance is the possibility of a frontal sinus being so rudimentary that from a clinical standpoint it is absent, hence there would be a greatly added risk of injuring the cribriform plate in trying to pass probes or sounds into the sinus.

While emphasising the assistance afforded me by skiagraphy, I am especially indebted to Mr. James Taylor for the skiagrams of a large number of cases at the Bristol Royal Infirmary, including those examples which have been used for the illustrations included in this article.

For my method of operating by the anterior route the essential instruments are (1) small angular punch forceps, made in two sizes, (2) frontal sinus rasps for the crista nasalis. For the rapid reduction of the nasal crest the

FIG. 5.

*The author's small raspatory. At the tip it measures only 2 mm. in width, and it can only cut forwards; the end also is blunt.*

guarded electric rotating burr is exceedingly useful, and I have also used with advantage sliding punch forceps, but neither of these instruments are essential. The bougies or sounds are merely for measuring the size of the fronto-nasal passage obtained, never for making the passage, but they are useful subsequently for maintaining the patency of the passage during the process of healing.

FIG. 6.

### The Operative Technique.

*A. Intranasal operation.*

1. With small angular ethmoidal forceps engage the anterior margin of the middle turbinal at its point of attachment to the outer nasal wall. Cutting through this, the forceps enter the anterior ethmoidal cells in front of the fronto-nasal passage (*see* fig. 7).

2. Keeping to the outer side of the vertical plate of the ethmoid, clip away all the agger cells and the other anteconchal cells right up to the crista nasalis (fig. 8).

3. The anterior ethmoidal cells lying behind or above the fronto-nasal duct, including the bulla ethmoidalis, are now removed by the forceps as far back as may be necessary.

4. Using the larger forceps, the thicker projecting partitions of the cells are laid open and punched away. (Only the blunt tip of the female blade can come in contact with the roof.)

5. The bougies (fig. 6) are then passed into the sinus, so as to gauge the size of the fronto-nasal channel thus formed. Usually Nos. 18 or 19 will enter, sometimes 19–23, or 19–25. (The figures give the circumference in millimetres, hence 19 has a diameter of 6.05 mm., or $\frac{1}{4}$ in. A 19–25 bougie measures 6 mm. in width and 8 mm. in antero-posterior diameter.)

In a considerable percentage of cases this intranasal operation suffices for permanent free and spontaneous drainage of the sinus.

*B. Pernasal operation.*

If such a large bougie will not enter, the bone corresponding to the nasal crest *may* be shaved away by the sliding cutting forceps (fig. 9) till these large sizes can be introduced, or the crest reduced first by the smaller guarded burr, or a small-sized sharp raspatory (fig. 5), till the passage admits the burr or forceps. When a No. 17 enters the sinus the bony boss can be burred away first with the 4 mm. wide burr until it enters the sinus (fig. 10). When the frontal sinus opening lies well to the outer side and tends to guide entering probes towards the orbital roof, unless contra-indicated by skiagram, it is well to draw the sliding forceps or burr towards the front so as to enlarge the frontal ostium to the front and inwards rather than towards the orbital roof outwards.

6. With the small forceps, which now enter freely, the projecting walls of any remaining ethmoidal cells may be clipped away to render the passage more free.

Up to this point the middle turbinate body has been left practically intact, and the whole operative procedure takes place outside the vertical plate of the turbinal, that is outside the line of the cribriform plate. In exceptional cases, where the anterior end of the turbinal is enlarged or cellular, it may be necessary to remove it at this stage, but as a rule it is left for some days, at least until the sinus has been lavaged several times. This has the advantage of shutting off the operative field, through which the infective purulent discharges of the sinus have to make their exit, from the olfactory fissure. If one makes an entry to the fronto-ethmoidal cells through the upper part of the vertical plate of the middle turbinal, not far from the cribriform plate, in the manner described by Mosher, it involves the risk of a septic thrombo-phletitis, and increases the liability to a meningitis spreading by this route.

One advantage that may be claimed for this method of entry as compared with methods with unguarded burrs is that the mucous membrane is not stripped from the posterior and lateral walls of the new passage, for although the cell partitions are clipped away, the mucous membrane of the cell bottom is mostly retained. Only anteriorly is the bone

Fig. 7.

*To show the initial point of entry in the intranasal frontal sinus operation. The small spheno-ethmoidal forceps are seen cutting the point of attachment of the middle turbinal to the outer nasal wall, thus entering the fronto-ethmoidal cells.*

Fig. 8.

*To show the author's small spheno-ethmoidal forceps clipping down walls of the ethmoidal cells external to the vertical plate of the middle turbinal.*

FIG. 9.

*The sliding cutting forceps reducing the crista nasalis and projecting bony ridges after the cells have been opened by the small cutting forceps. The middle turbinal shown in situ. The dotted line shows the forward extent of the cribriform plate.*

FIG. 10.

*Skiagram of patient showing 4 mm. burr on nasal crest.*

FIG. 11.

*Skiagram showing 6 mm. thick round bougie entering frontal sinus, but carried back so as to impinge against tabula interna because the nasal crest is thick.*

FIG. 12.

*The same case, but showing the 6 mm. (No. 19, French scale) bougie entering well into the sinus after reduction of the nasal crest by burring.*

*Skiagrams showing Watson-Williams's swan-head bougie, 8 mm. by 6 mm., entering the frontal sinus after removal of crista nasalis.*

FIG. 13.

*Case of W. C. Lateral view of left frontal sinus and swan-head oval bougie.*

FIG. 14.

*The same, antero-posterior aspect. The skiagram shows the right sinus healthy, the left sinus containing the bougie being diseased. (Skiagram reversed in print.)*

laid bare, and that for a strip about 6.5 mm. wide, and this can apparently recover itself by extension from the muco-periosteum on either side. Is it not better to have a 6 or 7 mm. wide passage which is lined with mucous membrane than a wider one which can only granulate over and which is much more liable to subsequent contraction ?

A septal deflection, unless so pronounced and so situated that it is impossible to reach the operative field, should be left to be dealt with when the infected sinuses have recovered or are more healthy ; if necessary, the septum can be pushed over to the other side by a Killian speculum. I have operated successfully on a frontal sinus when a septal deflection concealed from sight the middle turbinal and every part of the operative field.

After-treatment consists :—

1. In lavage of the sinus, first with saline solutions and weak peroxide of hydrogen and some mild antiseptic, such as colloidal or other silver preparations, iodine solutions, and so forth, and later with stronger solutions if necessary.

2. In the passage of the largest bougie the canal will take comfortably, repeated at short intervals to prevent adhesions, and to insure the passage remaining widely open until the sinus has become healthier, or the discharges disappear.

3. The use of vaccines, etc., has to be considered. But in cases of streptococcal infection it is always safer to give 30 to 50 c.c. of polyvalent antistreptococcic serum immediately before operating, and follow with sensitised vaccines.

The pernasal operation is a very much less serious procedure than the external radical operation, so that one cannot fairly contrast statistics. In the first place, an operation which, at the outside, usually involves only discomfort, no very appreciable risk, and no chance of a cosmetic deformity, is of course applicable to cases where the symptoms are altogether too slight to justify an external operation, hence the cases of internal operation cannot be fairly contrasted with those relatively rare and always severe conditions which alone justify resort to radical operation. On the other hand, a radical operation cannot be termed successful unless the nasal discharge clear up entirely; but an internal operation which overcomes the headaches and leaves the patient in good health is successful, though some amount of discharge persists, perhaps even sufficient to call for occasional lavage. And as nearly every patient who has had the internal operation described can lavage the frontal sinuses without more difficulty than is the case with a maxillary antral operation, the need for lavage is no hardship. But a large proportion of the patients lose the discharge altogether in course of time, and become as completely cured as the most successful case of radical external operation.

## Figures and Tables

**Fig. 2. f1:**
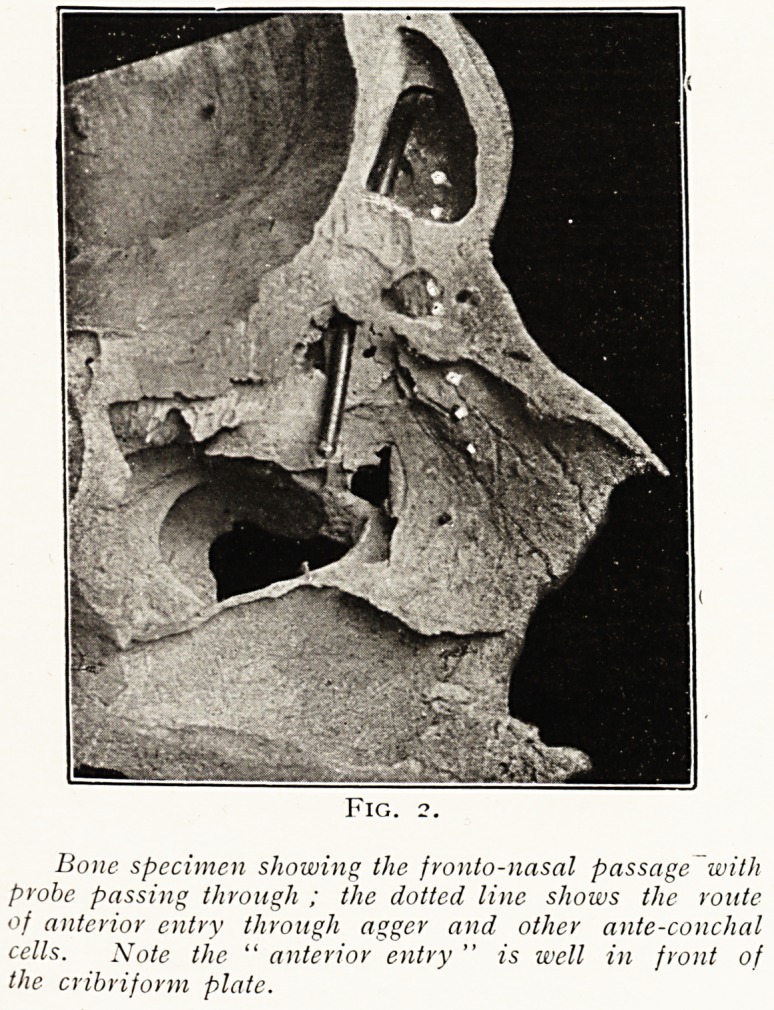


**Fig. 3. f2:**
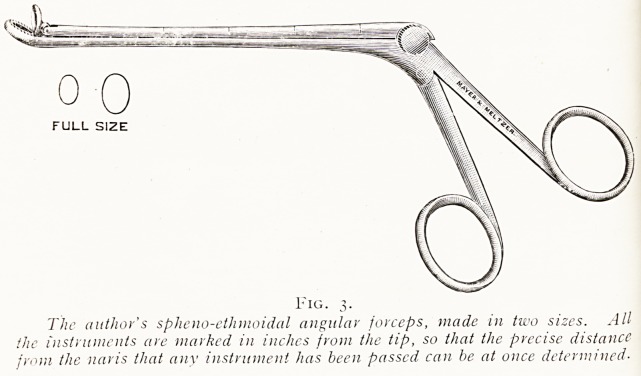


**Fig. 4. f3:**
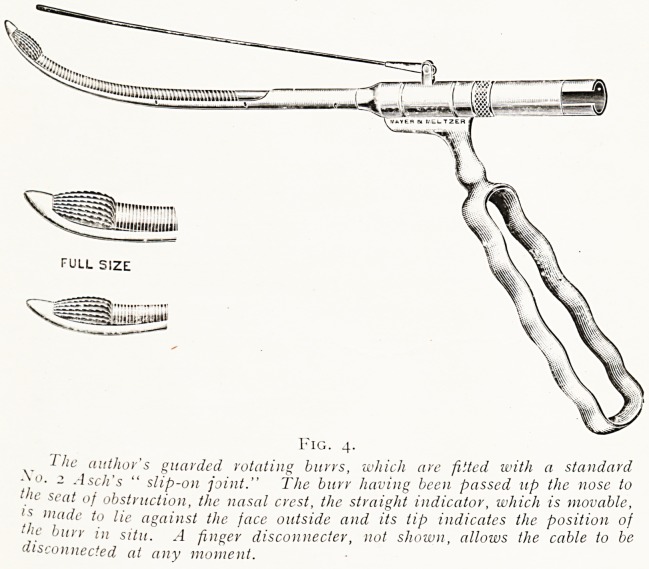


**Figure f4:**
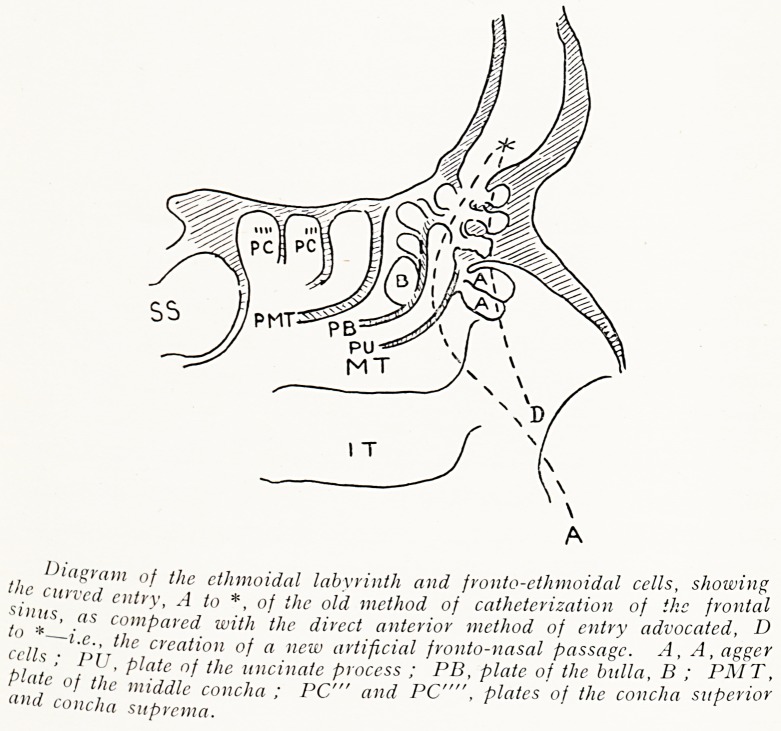


**Fig. 5. f5:**
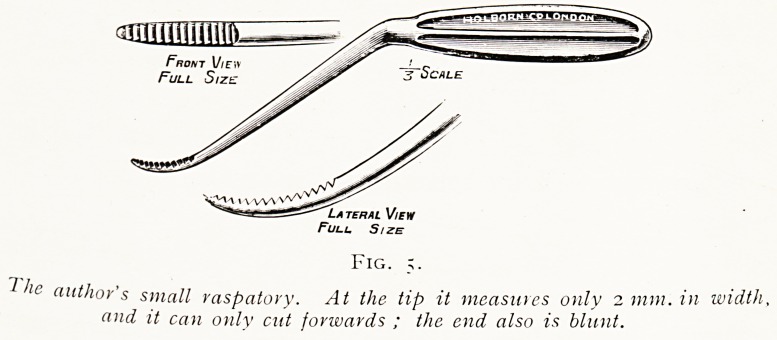


**Fig. 6. f6:**
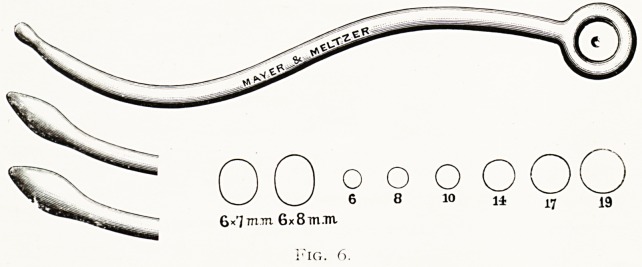


**Fig. 7. f7:**
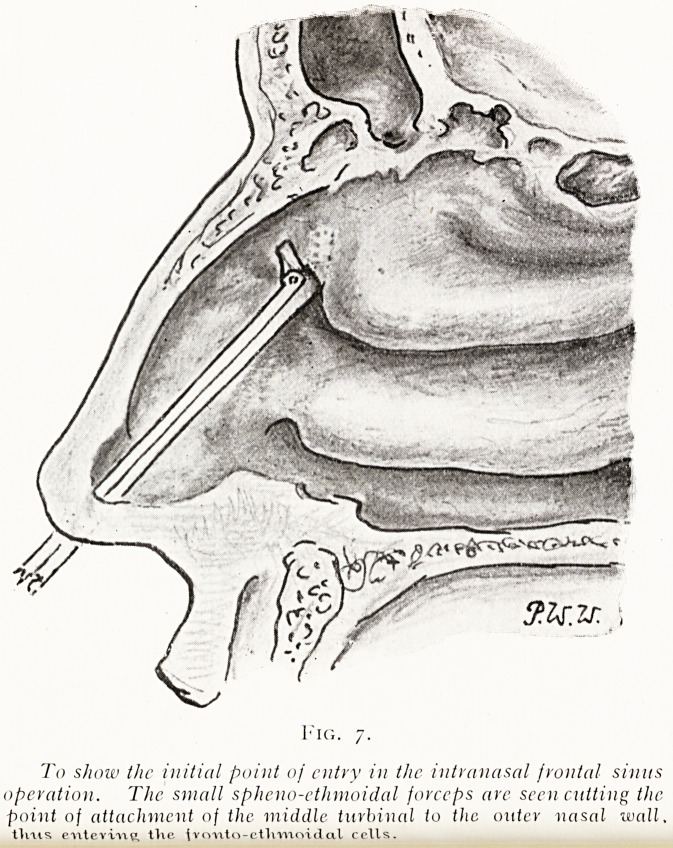


**Fig. 8. f8:**
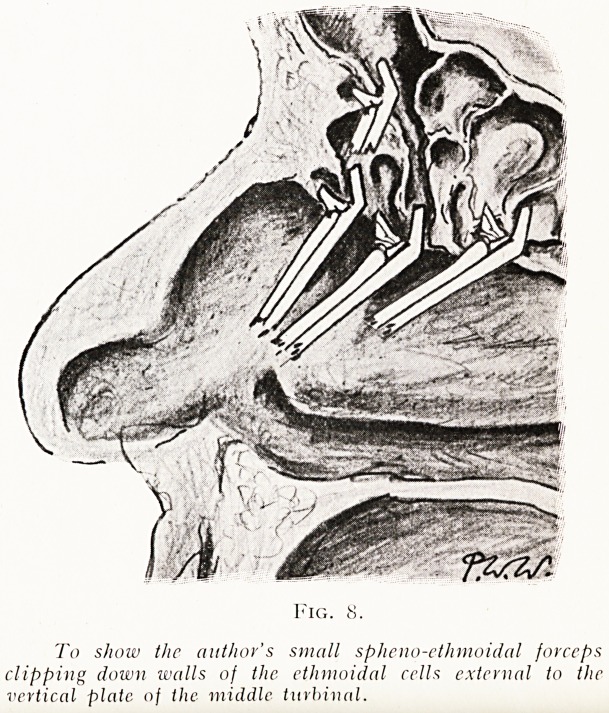


**Fig. 9. f9:**
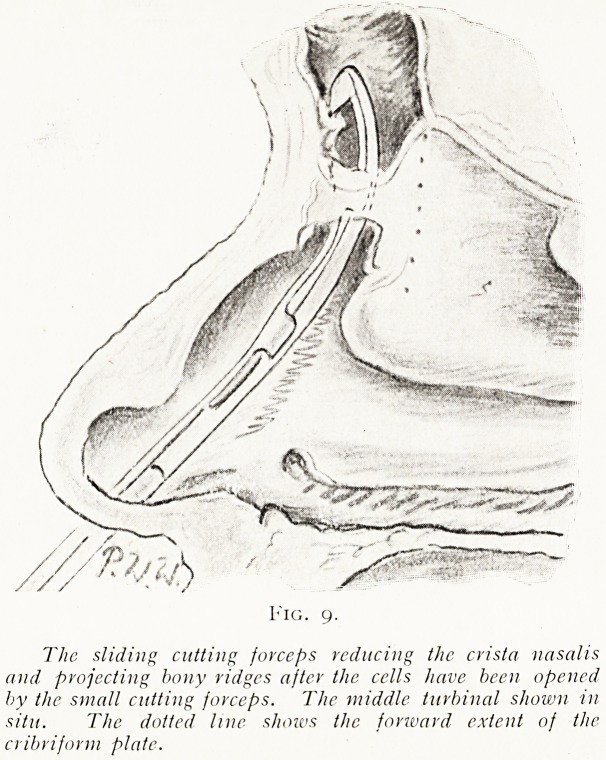


**Fig. 10. f10:**
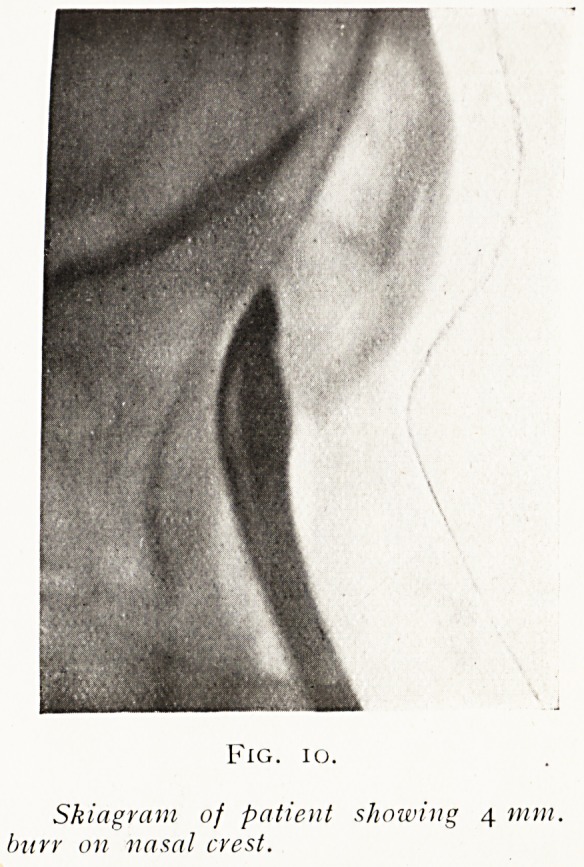


**Fig. 11. f11:**
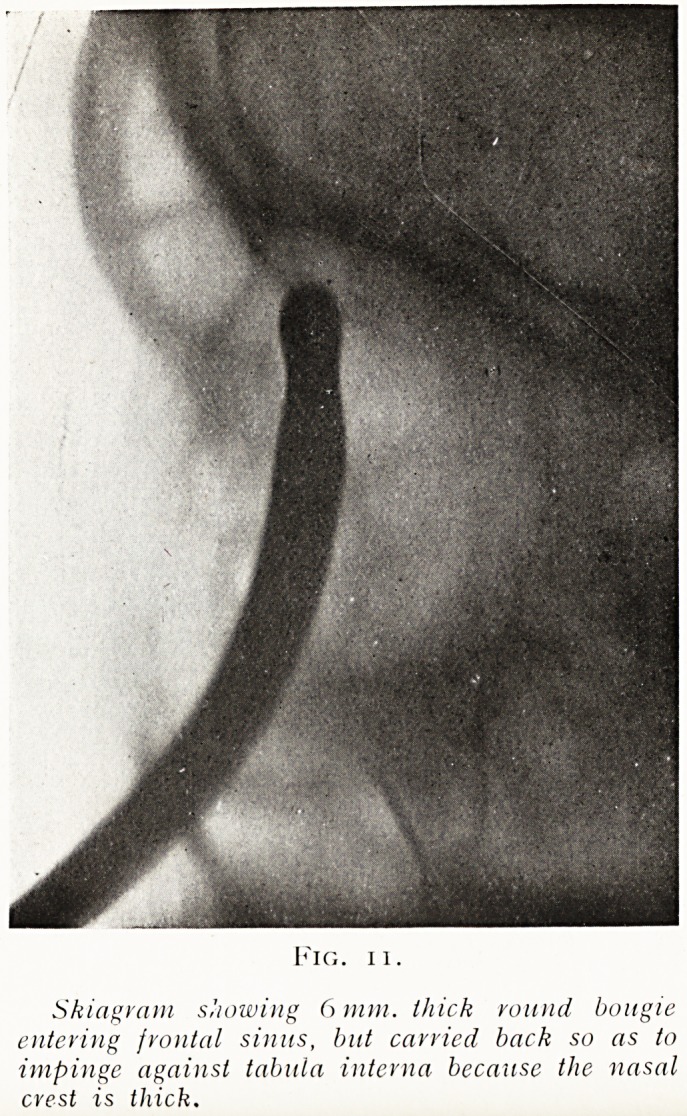


**Fig. 12. f12:**
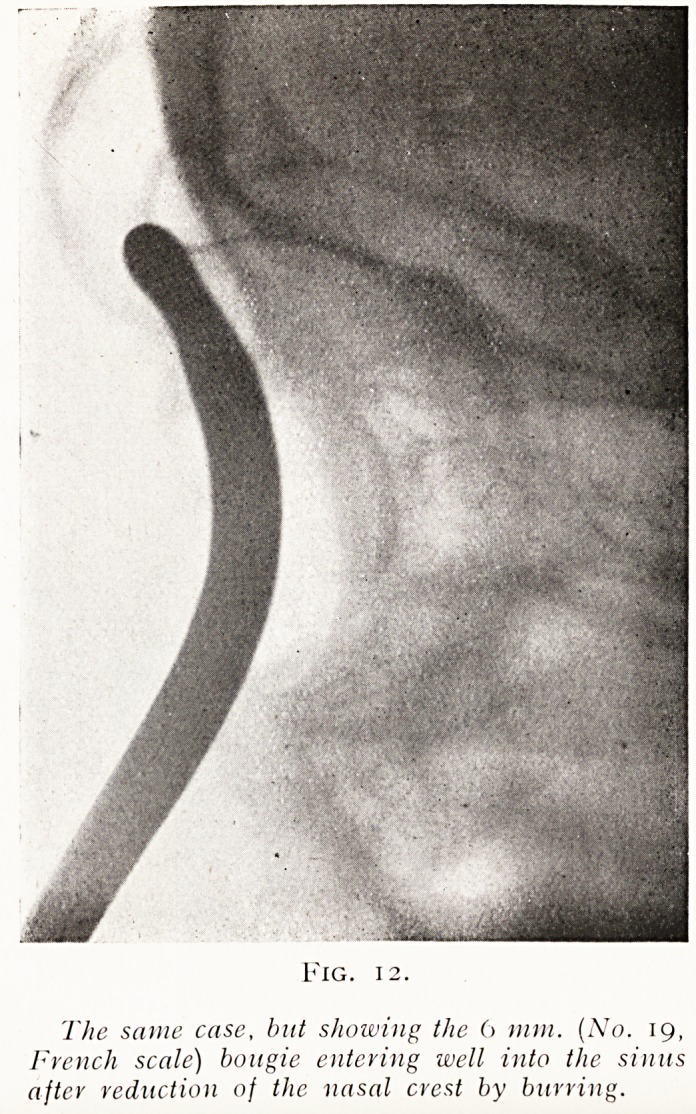


**Fig. 13. Fig. 14. f13:**